# Supplementary material for: Macrophage-released ADAMTS1 promotes muscle stem cell activation
Source: Nat Commun. 2017 Sep 22;8:669. doi: 10.1038/s41467-017-00522-7 (PMC5610267; doi:10.1038/s41467-017-00522-7)
Supplement: Supplementary file 1 — Supplementary Information [file 41467_2017_522_MOESM1_ESM.pdf]

File name: Supplementary Information
Description: Supplementary figures and supplementary table.

File name: Peer review file
Description:

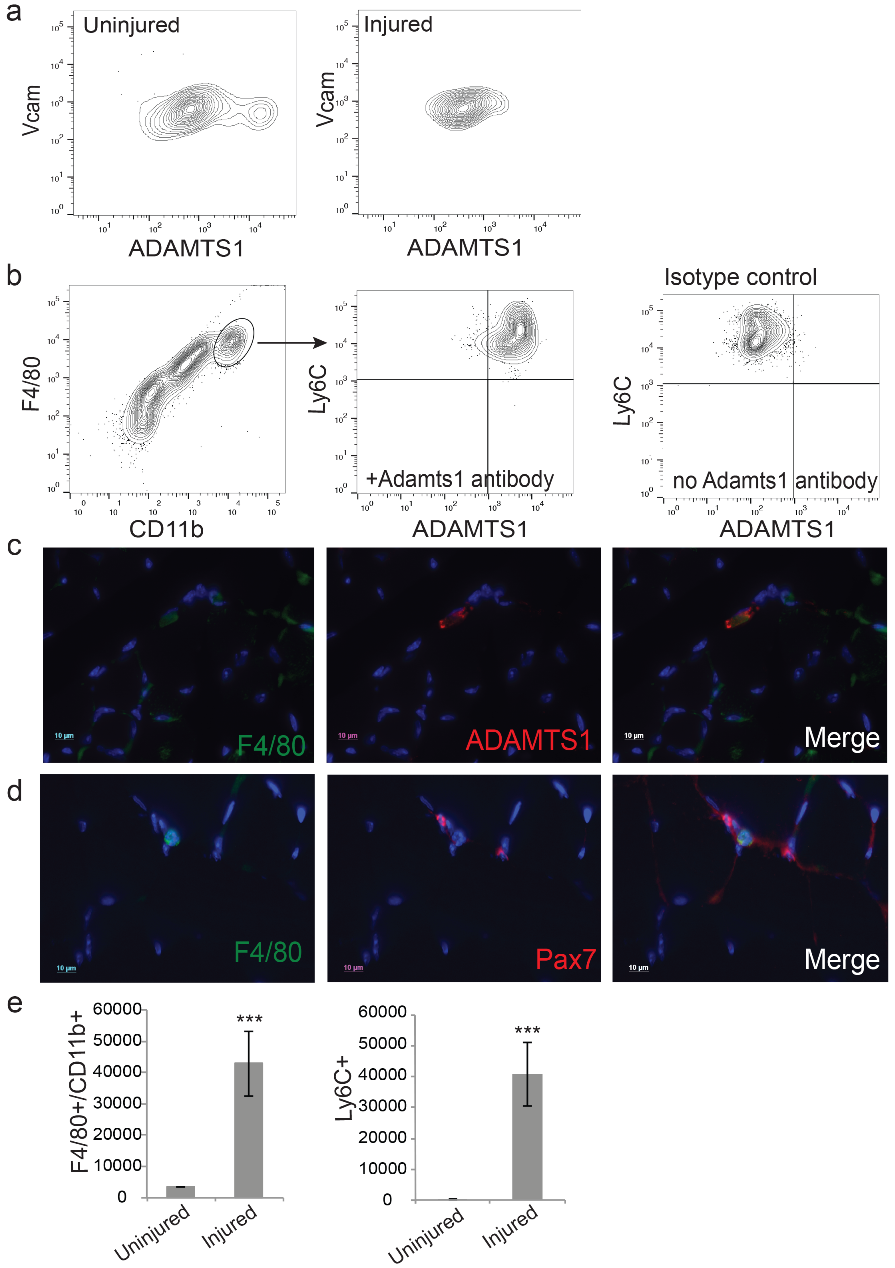

Supplementary Figure 1: Adamts1 is expressed in macrophages

(**a**) Representative flow cytometry plots measuring ADAMTS1 levels in satellite cells from uninjured compared to injured (day 1 post-injury) TA muscle showing that ADAMTS1 is not induced in satellite cells in response to injury. (**b**) Representative flow cytometry plots validating the ADAMTS1 antibody used for measuring ADAMTS1 expression by comparing ADAMTS1 levels in macrophages using the ADAMTS1 antibody compared to isotypic negative control antibody. A gate set at 10^3 distinguishes ADAMTS1 positive and negative populations. (**c**) Images of IHC performed on histological sections of wild-type mice prepared from frozen cryosections of uninjured TA muscle and stained using antibodies against ADAMTS1 (red) and F4/80 (green). (**d**) Images of IHC performed on histological sections of wild-type mice prepared from frozen cryosections of uninjured TA muscle and stained using antibodies against Pax7 (red) and F4/80 (green). (**e**) Absolute counts of total macrophages (left) and Ly6C+ macrophages (right) in uninjured (n= 4) compared to injured (n=4) (day 1 post-injury) TA muscle by flow cytometry. ****P* <0.001. Error bars represent standard deviations. Statistical significance tested using paired *t*-tests.

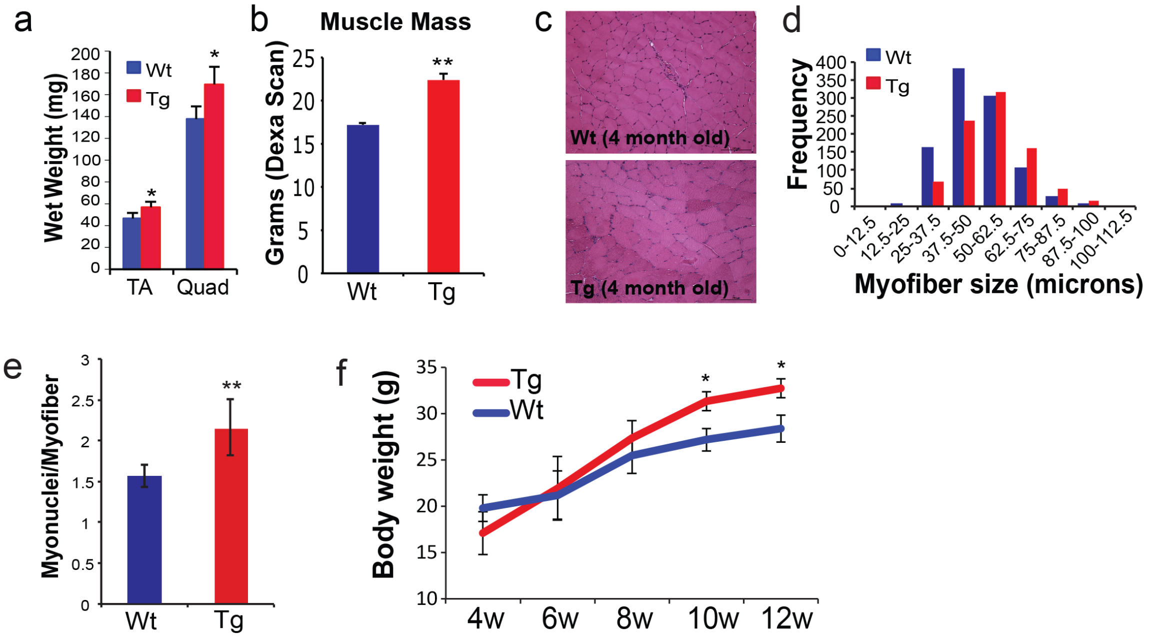

Supplementary Figure 2: Adamts1 mice have accelerated postnatal muscle growth.

(**a**) Gross wet weights of TA and Quad muscles from Wt (*n*=5) and Tg (*n*=3) mice at 4-month of age. (**b**) Quantification of lean body mass in Wt (*n*=6) and Tg (*n*=6) using dual energy X-ray absorption (DEXA). (**c**) Images of histological sections of TA muscles isolated from 4-month old Wt and Tg mice and stained with H&E. Scale bar= 100μm (**d**) Quantification of the cross-sectional diameters of myofibers from the TA muscles of 4-month old Wt (*n*=3) and Tg (*n*=4) mice demonstrating that the myofibers in Tg mice are larger than Wt. (**e**) Quantification of the number of myonuclei per myofiber in Tg compared to control mice (*n*=3 mice per genotype, >200 myofibers per mouse were counted) (**f**) Serial total body weights of Wt (*n*=9) and Tg (*n*=6) mice. *P <0.05, **P <0.01, ****P <0.0001. Error bars represent standard deviations. Statistical significance tested using paired *t*-tests.

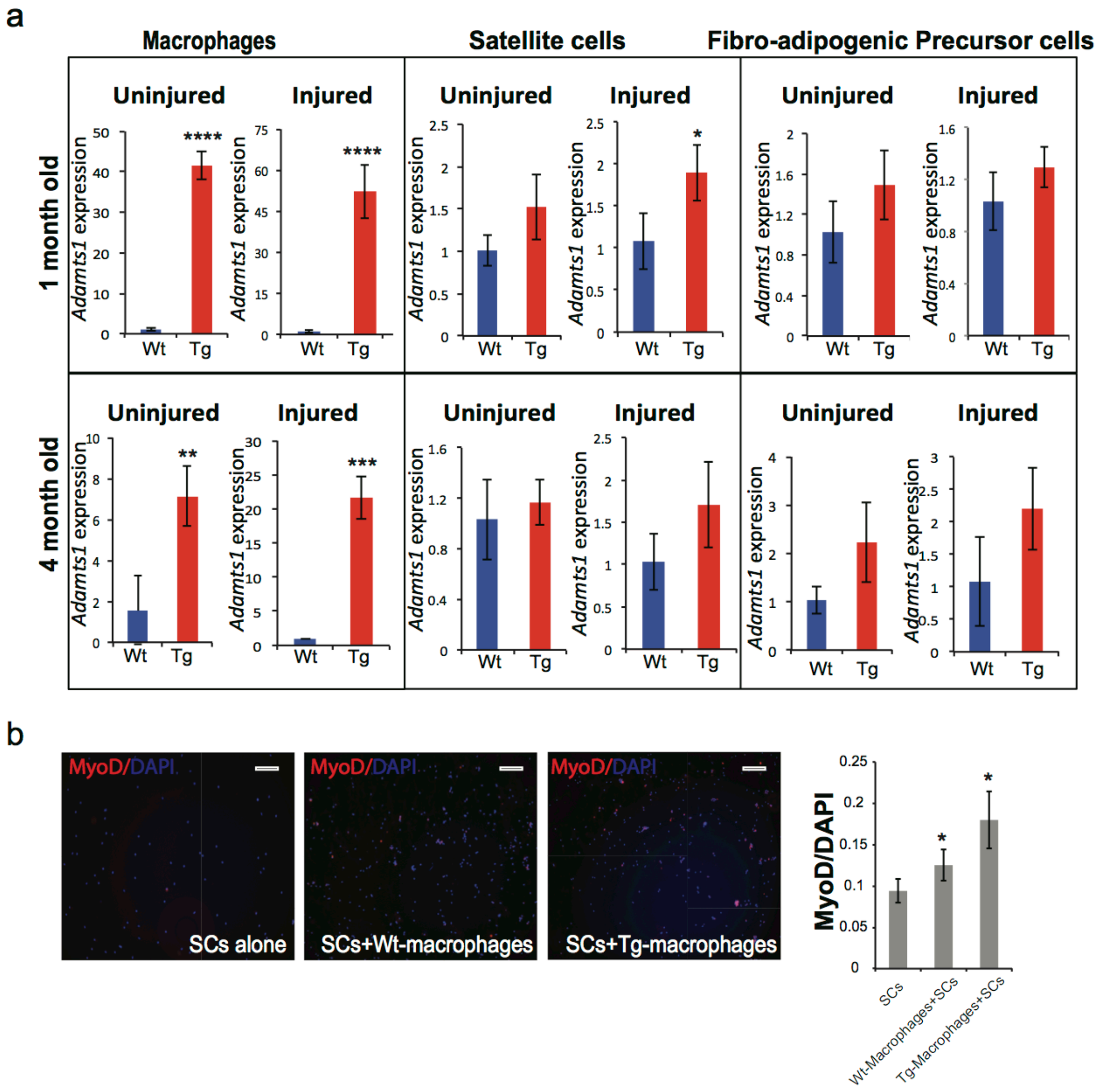

Supplementary Figure 3: Overexpression of *Adamts1* in macrophages from Adamts1 mice increases satellite cell activation.

(**a**) RT-qPCR quantifying the level of *Adamts1* expression in FACS isolated macrophages, satellite cells and fibro-adipogenic precursor cells from uninjured and injured (day 1 post-injury) TA muscles in Adamts1 (Tg) and wild-type (Wt) littermate mice at 1-month and 4-months of age. (**b**) (left) images of ICC of 10^4 satellite cells FACS isolated from wild-type mice and cultured alone or co-cultured with 10^4 macrophages from Wt or Tg mice (*n*=3 per genotype) for 12 hrs (Scale bar= 100μm) and stained for MyoD to detect activation. (right) Quantification of the number of MyoD+ SC per high-powered field.

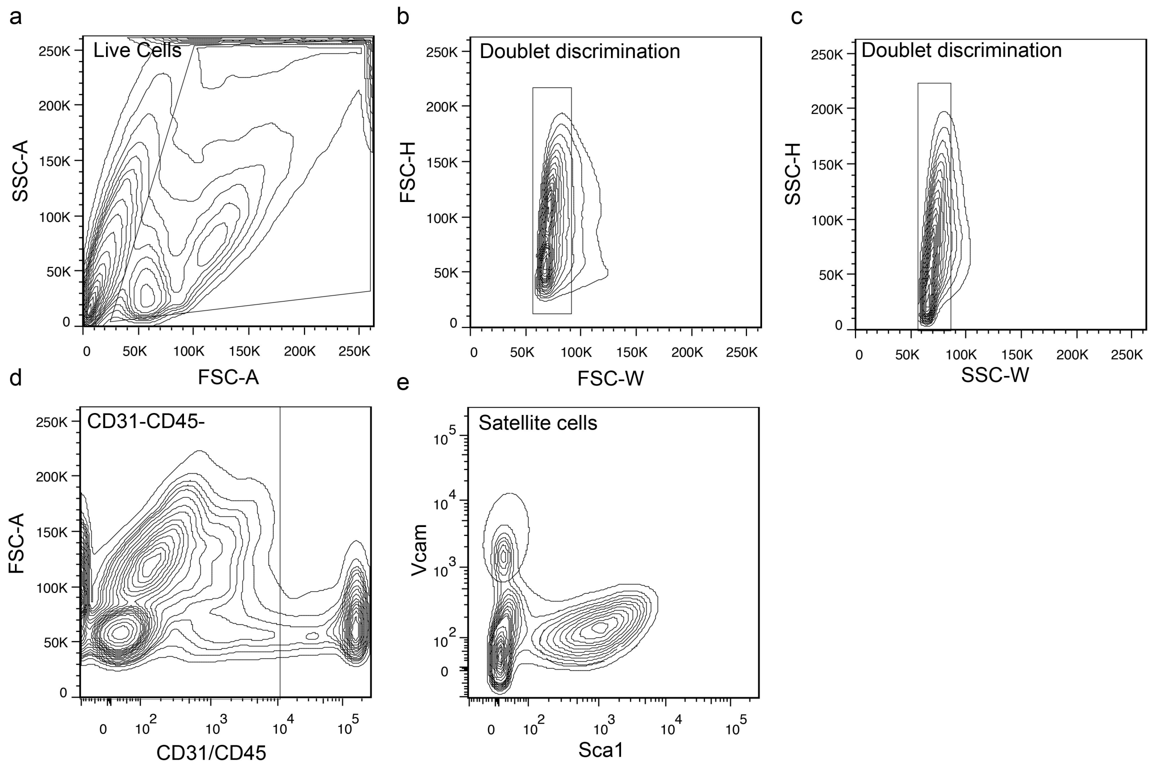

Supplementary Figure 4: FACS isolation of satellite cells

Satellite cells were purified from collagenase digested muscle tissue using FACS by gating on forward and side scattered cells (**a-c**) to isolate live single cells followed by gating cells that were $CD31^-$ and $CD45^-$ (**d**) as well as $Vcam^+$ and $Sca1^-$ (**e**).

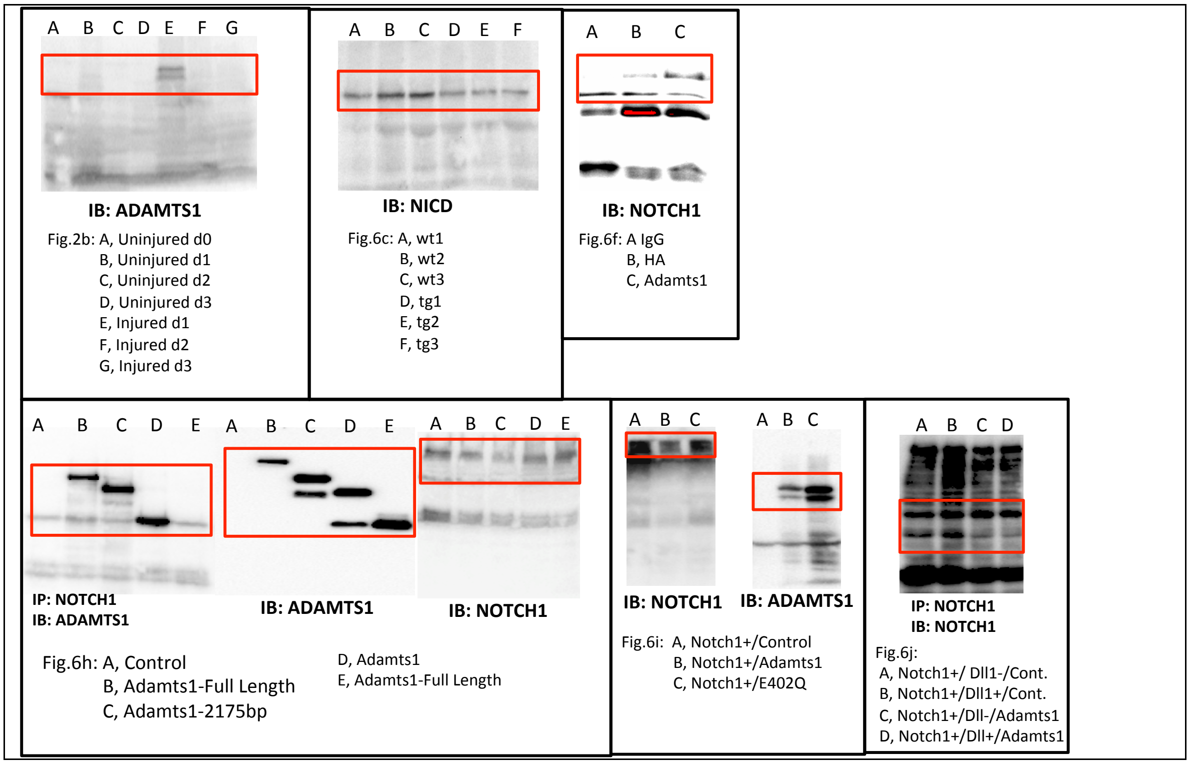

Supplementary Figure 5: Uncropped immunoblots of main figures.

Corresponding main figures are indicated below each blot.

Gene	Primer Sequence
Hes-1	F: 5’ -ATCATGGAGAAGAGGCGAAG- 3’<br>R: 5’ -CAGGTTCCGGAGGTGCTT- 3’
Hey-1	F: 5’ -CCGACGAGACCGAATCAATA- 3’<br>R: 5’ -CCTGCAGTGTGCAGCATTTT- 3’
Adamts1 Transgene	F: 5’ -TTAATGGACACCCTGCTTCC- 3’<br>R: 5’ -ATCTTCCCATTCTAAACAACACCC- 3’
Adamts1	F: 5’ -GGGAATGAGCCCACTGTAGA- 3’<br>R: 5’ -CACAGCCAGCTTTCACACAC- 3’
Notch1	F: 5’ -ACTGGTCCCCACTGTGAACT- 3’<br>R: 5’-CCCCATTCTTGCAGTTGTTT- 3’
Rpl19	F: 5’ -GGATCCCAATGAGACCAATG- 3’<br>R: 5’ -TCTCCTCCTCCTTGGACAGA- 3’

Supplementary Table 1: Primer sequences

Table of the sequence of primers used for RT-qPCR.
